# Murine neonatal cardiac B cells promote cardiomyocyte proliferation and heart regeneration

**DOI:** 10.1038/s41536-023-00282-7

**Published:** 2023-02-11

**Authors:** Yong Tan, Xuewen Duan, Bo Wang, Xingguang Liu, Zhenzhen Zhan

**Affiliations:** 1 Department of Liver Surgery, Shanghai Institute of Transplantation, Renji Hospital, Shanghai Jiao Tong University School of Medicine, Shanghai, 200127 China; 2 Key Laboratory of Arrhythmias of the Ministry of Education of China, Research Center for Translational Medicine, Shanghai East Hospital, Tongji University School of Medicine, Shanghai, 200120 China; 3 Department of Pathogen Biology, Naval Medical University, Shanghai, 200433 China

**Keywords:** Cell division, Cardiomyopathies, Cell death and immune response, Experimental models of disease

## Abstract

The irreversible loss of cardiomyocytes in the adult heart following cardiac injury leads to adverse cardiac remodeling and ventricular dysfunction. However, the role of B cells in cardiomyocyte proliferation and heart regeneration has not been clarified. Here, we found that the neonatal mice with B cell depletion showed markedly reduced cardiomyocyte proliferation, leading to cardiac dysfunction, fibrosis scar formation, and the complete failure of heart regeneration after apical resection. B cell depletion also significantly impaired heart regeneration and cardiac function in neonatal mice following myocardial infarction (MI). However, B cell depletion in adult mice suppressed tissue inflammation, inhibited myocardial fibrosis, and improved cardiac function after MI. Interestingly, B cell depletion partially restricted cardiomyocyte proliferation in adult mice post-MI. Single-cell RNA sequencing showed that cardiac B cells possessed a more powerful ability to inhibit inflammatory responses and enhance angiogenesis in the postnatal day 1 (P1) mice compared with P7 and adult mice. Besides, the proportion of cardioprotective B cell clusters with high expression levels of *S100a6* (S100 calcium-binding protein A6) and *S100a4* (S100 calcium-binding protein A4) was greatly decreased in adult heart tissues compared with neonatal mice after cardiac damage. Thus, our study discovers that cardiac B cells in neonatal mice are required for cardiomyocyte proliferation and heart regeneration, while adult B cells promote inflammation and impair cardiac function after myocardial injury.

Because the proliferative capacity of cardiomyocytes is extremely limited in adult mammalian hearts, the irreversible loss of cardiomyocytes following cardiac injury markedly impairs cardiac function, leading to adverse cardiac remodeling and heart failure. However, early neonatal mice have a strong ability in cardiomyocyte proliferation and cardiac regeneration after heart damage such as apical resection (AR)^1^. Besides cardiomyocytes, non-myocytes in heart tissue also play different roles in the cardiac repair process^2^. Previous studies showed that cardiac macrophage depletion led to hyper-fibrosis and inhibited neovascularization after heart injury in neonatal mice. Transplantation of neonatal mouse cardiac macrophages into myocardial infarction (MI)-injured adult mice enhanced cardiomyocyte proliferation and facilitated cardiac repair^3,4^. The regulatory T cells (Treg) were reported to promote myocardial regeneration in a paracrine manner, while CD4^+^ Th1 and Th17 cells directly inhibited the proliferation and promoted the apoptosis of neonatal cardiomyocytes^5^. However, the roles of other cardiac immune cells in cardiac regeneration remain to be elucidated. B cells are a type of prominent immune cells in the injured heart; here we discovered the indispensable function of cardiac B cells in improving cardiomyocyte proliferation and heart regeneration in neonatal mice.

First, we found that the proportion of CD45^+^CD19^+^ B cells in cardiac tissues gradually increased at day 0–7 after AR of one-day-old mice (Supplementary Fig. 1a, b). To investigate the role of B cells in neonatal cardiac regeneration, we utilized Cd19-e(IRES-DTREGFP)2 (simplified as Cd19^DTR^) mice with knock-in of diphtheria toxin (DT) receptor in stop codon of *Cd19* gene (Supplementary Fig. 1c). CD19^+^ B cells in heart, spleen, and blood of Cd19^DTR^ mice were almost completely depleted after three times of intraperitoneal DT injection over 4 days after cardiac injury (Supplementary Fig. 1d–f). AR operation was performed on 2-day-old Cd19^DTR^ or wild type (WT) control mice with DT or phosphate-buffered saline (PBS) administration (Supplementary Fig. 1c). Masson’s trichrome staining showed that the marked fibrosis scar of myocardium occurred in neonatal Cd19^DTR^ mice with B cell depletion at day 21 after AR, while the hearts of neonatal Cd19^DTR^ mice with PBS administration and WT control mice with PBS or DT administration could completely regenerate without scar post-AR, indicating that the neonatal mouse hearts with B cell depletion failed to regenerate and repair (Fig. 1a). Furthermore, the cardiac function including the percentages of left ventricular ejection fraction (LVEF) and left ventricular fractional shortening (LVFS) were significantly reduced in Cd19^DTR^ neonatal mice at day 21 after AR with DT administration as compared with PBS administration (Fig. 1b). MI was also performed on two-day-old Cd19^DTR^ mice with DT or PBS administration and heart regeneration was examined at 3 weeks after MI (Supplementary Fig. 1c). Similar to AR model, B cell depletion could suppress heart regeneration, resulting in scar formation and impaired cardiac function post-MI in neonatal mice (Fig. 1c, d). We then explored whether B cell depletion affected cardiomyocyte proliferation in neonatal mice. Immunofluorescence staining showed that the proliferated cardiomyocytes with EdU-, pH3-, or Ki67-positive expression were dramatically decreased at day 7 after AR in Cd19^DTR^ mice with DT administration (Fig. 1e–g). These data indicate that B cells promote cardiomyocyte proliferation and enhance cardiac regeneration in neonatal mice, leading to improved cardiac function after myocardial damage.

**Fig. 1:**
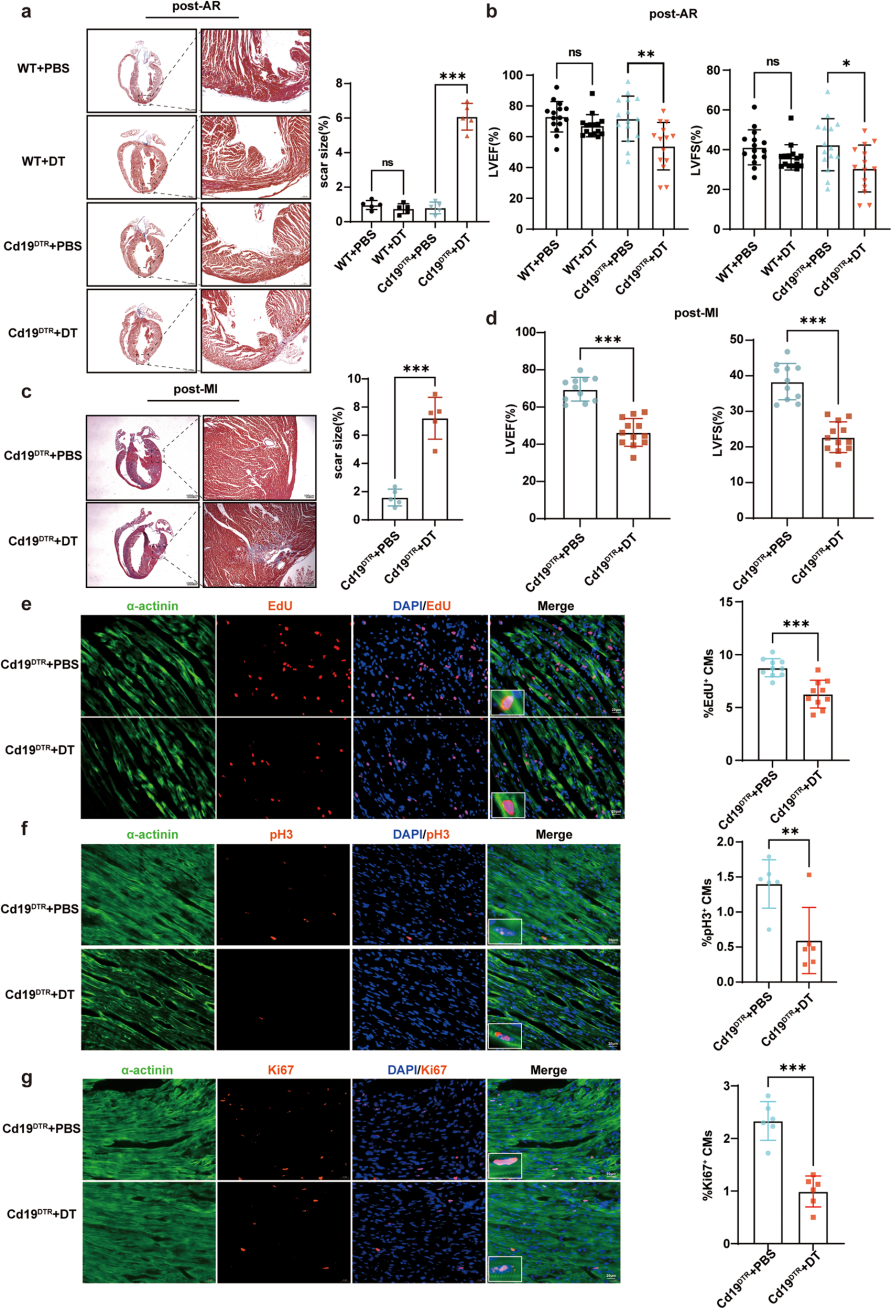
Murine neonatal cardiac B cells promote cardiomyocyte proliferation and heart regeneration. **a** Representative images and quantification of heart sections stained with Masson’s trichrome from the neonatal CD19^DTR^ or WT mice with DT or PBS administration 3 weeks after AR. Scale bars, 1000 μm (left) or 100 μm (right). Each symbol in quantification represents one mouse (*n* = 5 mice for each group, one-way ANOVA). **b** Echocardiographic measurement of LVEF and LVFS of the neonatal CD19^DTR^ or WT mice with DT or PBS administration 3 weeks after AR. Each symbol represents one mouse (*n* = 14 or 15 mice for each group, one-way ANOVA). **c** Representative images and quantification of heart sections stained with Masson’s trichrome from the neonatal CD19^DTR^ mice with DT or PBS administration 3 weeks after MI. Scale bars, 1000 μm (left) or 100 μm (right). Each symbol in quantification represents one mouse (*n* = 5 mice for each group, unpaired Student’s *t*-test). **d** Echocardiographic measurement of LVEF and LVFS of the neonatal CD19^DTR^ mice with DT or PBS administration 3 weeks after MI. Each symbol in quantification represents one mouse (*n* = 11 or 12 mice for each group, unpaired Student’s *t*-test). **e**–**g** Representative immunofluorescent staining images and quantification of EdU^+^, pH3^+^, or Ki67^+^ cardiomyocytes from myocardial tissues of the neonatal CD19^DTR^ mice with DT or PBS administration at day 7 post-AR. Scale bars, 20 μm. Each symbol in quantification indicates one representative image from one heart section of one mouse (*n* = 10 mice for each group in **e**; *n* = 6 mice for each group in **f** and **g**; unpaired Student’s *t*-test). The data were shown as mean ± SD. *p < 0.05. **p < 0.01, and ***p < 0.001.

Next, we investigated whether cardiac B cells in adult mice functioned in heart repair and cardiomyocyte proliferation of injured myocardium. The markedly increased cardiac function was found in adult Cd19^DTR^ mice (8–10-week-old) with DT administration as compared with adult Cd19^DTR^ mice with PBS administration and adult WT mice with PBS or DT treatment after MI (Supplementary Fig. 2a, b). B cell depletion mediated by DT administration reduced cardiac fibrosis and scar size of adult Cd19^DTR^ mice post-MI, which partially attributed to the decreased expression of fibrosis-related genes (such as fibronectin 1 [*Fn1*] and collagen type I alpha 1 chain [*Col1a1*]) and the limited myofibroblast proliferation (Supplementary Fig. 2c–e). Besides, DT injection did not influence the survival rate of adult Cd19^DTR^ mice following MI, compared with PBS-treated adult Cd19^DTR^ mice (Supplementary Fig. 2f). The mRNA expression levels of inflammatory factors including *Il6*, *Il1β*, C-C motif chemokine ligand 5 (*Ccl5*) and C-X-C motif chemokine ligand 10 (*Cxcl10*) were significantly inhibited in cardiac tissues of DT-treated adult Cd19^DTR^ mice post-MI (Supplementary Fig. 2g), which consisted with the previous research that B cell depletion in adult mice with anti-CD20 antibody alleviated myocardial injury by suppressing pro-inflammatory factor production and monocyte infiltration after MI^6^. It is also reported that enhanced B cell responses promoted cardiac fibrosis and aggravated myocardial damage^7^. However, EdU-positive cardiomyocytes in DT-treated adult Cd19^DTR^ mice were less than that in Cd19^DTR^ mice with PBS administration at day 7 post-MI (Fig. 2a), indicating that adult cardiac B cells also possessed some ability in promoting cardiomyocyte proliferation in adult mice. We further re-analyzed single-cell RNA sequencing (scRNA-Seq) data of adult mouse heart tissues after MI or sham operation presented in previous research (GSE163465)^8^. Several genes encoded secreted proteins that had been reported to promote cell proliferation were significantly upregulated in B cells from cardiac tissues of adult mice post-MI, which included secretory leukocyte peptidase inhibitor (*Slpi*)^9^, immunoglobulin heavy constant gamma 1 (*Ighg1*)^10^, lipocalin 2 (*Lcn2*)^11^, s100 calcium-binding protein A8 (*S100a8*)^12^, s100 calcium-binding protein A9 (*S100a9*)^13^ and C-X-C motif chemokine ligand 2 (*Cxcl2*)^14^ (Fig. 2b). These results suggest that cardiac B cells from adult mice enhance cardiomyocyte proliferation after myocardial injury in a paracrine manner. Despite adult cardiac B cells also possessed some ability in promoting cardiomyocyte proliferation in adult mice, the very limited cardiomyocyte self-renewal enhanced by B cells was not sufficient to restore the markedly impaired cardiac function caused by the inflammation injury in the myocardium and cardiac fibrosis facilitated by B cells. Thus, B cell depletion in adult mouse heart improves cardiac function post-MI mainly through attenuating tissue inflammation injury and inhibiting cardiac fibrosis.

**Fig. 2:**
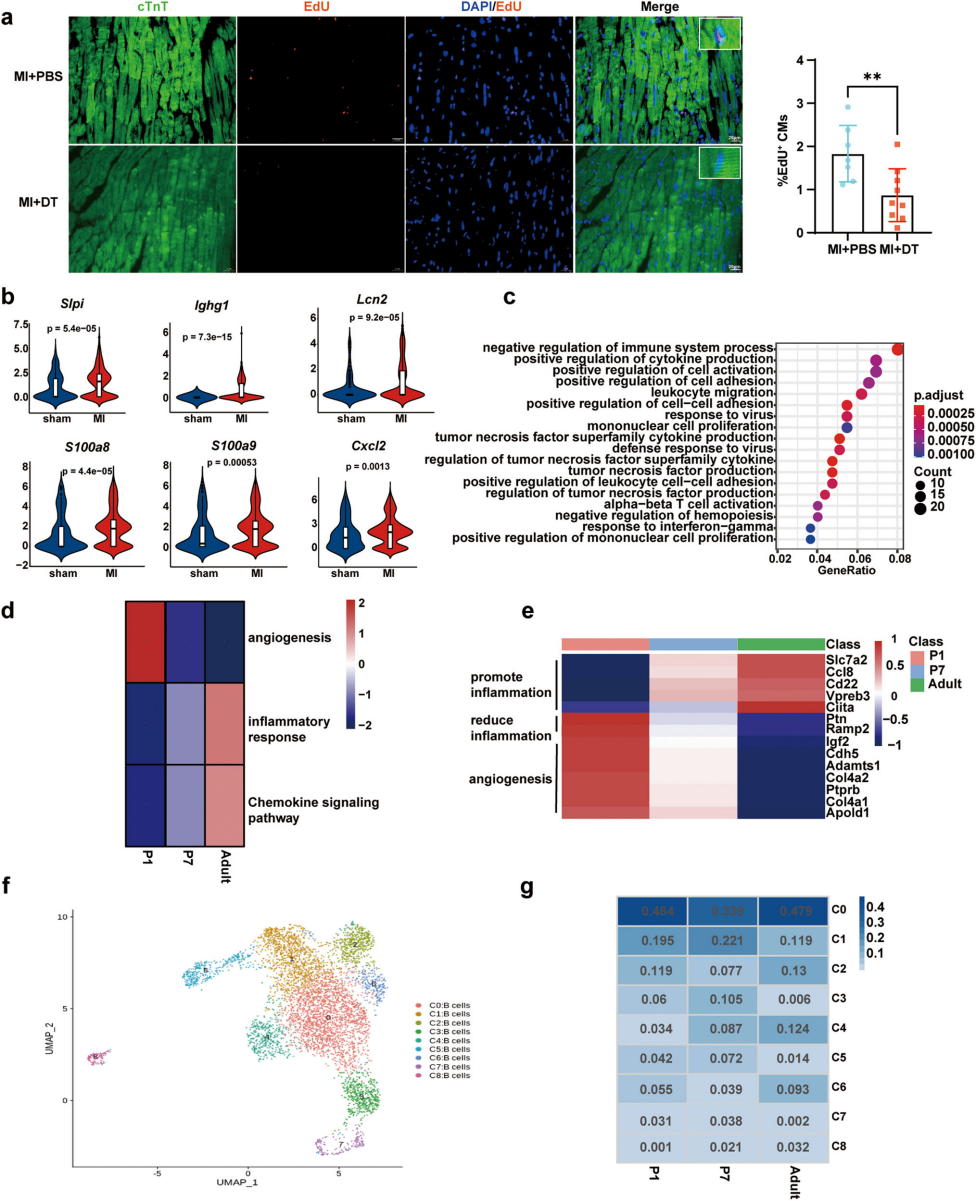
Murine neonatal cardiac B cells possess the greater ability to inhibit inflammatory responses and promote angiogenesis after cardiac injury. **a** Representative immunofluorescent staining images and quantification of EdU^+^ cardiomyocytes from myocardial tissues of adult CD19^DTR^ mice with DT or PBS administration at day 7 post-MI. Each symbol in quantification indicates one representative image from one heart section of one mouse (MI + PBS group, *n* = 7 mice; MI + DT group, *n* = 9 mice; mean ± SD; unpaired Student’s *t*-test). Scale bars, 20 μm. **b** Violin plots showing the expression scores of cell proliferation-associated genes (*Slpi*, *Ighg1*, *Lcn2*, *S100a8*, *S100a9*, and *Cxcl2*) in cardiac B cells from adult mice subjected to MI or sham operation (GSE163465). **c** GO enrichment analysis of DEGs in B cells from heart tissues at day 7 after AR operated on P1 and P7 mice. **d** Heatmap of DEGs enriched in angiogenesis, inflammatory responses, and chemokine signaling pathway of cardiac B cells prepared as in **c** and from adult mouse heart after MI (GSE163465). **e** Heatmap of top DEGs from cardiac B cells prepared as in **c** and from adult mouse heart after MI (GSE163465). **f** UMAP plots displaying B cell clusters in heart tissues prepared as in **c** and from adult mouse heart after MI (GSE163465). **g** Distribution of B cell proportions in each of the nine clusters from heart tissues prepared as in **c** and from adult mouse heart after MI (GSE163465). **p < 0.01.

To further elucidate the difference of B cell function in mice of different ages after myocardial injury, CD45^+^ cells isolated from heart tissues at day 7 after AR operated on the postnatal day 1 (P1) and 7 (P7) WT mice were applied to scRNA-Seq (Supplementary Fig. 3a). t-distributed stochastic neighbor embedding (t-SNE) plots showed 26 major cell populations in CD45^+^ cells of heart tissues from P1 and P7 WT mice (Supplementary Fig. 3b). Gene Ontology (GO) analysis showed that the differentially expressed genes (DEGs) enriched in negative regulation of immune system process of B cells from P1 hearts, compared with that in P7 hearts of WT mice after AR (Fig. 2c). Kyoto Encyclopedia of Genes and Genomes (KEGG) analysis found that the DEGs enriched in phagosome and lysosome of B cells in heart tissues of P1 mice post-AR (Supplementary Fig. 3c). These results reveal that the neonatal cardiac B cells in P1 mice display the higher capacity in inhibiting inflammatory responses and promoting clearance of cell debris after myocardial injury.

The combined analysis of our scRNA-Seq data and previously reported scRNA-Seq data (GSE163465) found that the progressively increasing capacity in promoting inflammatory responses and chemokine signaling was showed in cardiac B cells from P1 mice to adult mice after myocardial injury, whereas the neonatal cardiac B cells of P1 mice displayed the highest levels of anti-inflammatory genes such as pleiotrophin (*Ptn*) and receptor activity modifying protein 2 (*Ramp2*) (Fig. 2d, e). The heatmap results also showed the highest ability in enhancing angiogenesis in P1 cardiac B cells compared with P7 and adult cardiac B cells (Fig. 2d, e). Furthermore, the heterogeneity of B cells from hearts of P1, P7, and adult mice (GSE163465) were depicted by uniform flow shape approximation and projection (UMAP) and 9 major B cell subsets were identified by signature genes (Fig. 2f and Supplementary Fig. 3d). We found that the proportion of cardioprotective B cell subset with high expression levels of S100 calcium-binding protein A6 (*S100a6*) and S100 calcium-binding protein A4 (*S100a4*) (cluster 1) were greatly decreased in adult mice (GSE163465), compared with P1 and P7 mice after cardiac damage (Fig. 2g). Previous studies have shown that S100A4 and S100A6 can attenuate tissue damage and reduce cardiomyocyte death after MI^15^. Thus, the decrease of cardioprotective B cell subsets in adult mouse heart also may be a pathogenic factor that adult B cells lead to the aggravated cardiac injury of adult mice post-MI.

Macrophages were first reported to affect myocardial regeneration, with depletion of neonatal mouse macrophages preventing heart regeneration after myocardial injury. Distinct cardiac macrophage populations derived from embryonic and adult lineages are important determinants of tissue repair and inflammation, respectively^3,16^. Researchers found that innate immunity alone was insufficient for neonatal heart regeneration and depletion of Treg cells also failed to complete heart regeneration following myocardial injury^17^. Our study further discovers that B cells, as a type of prominent acquired immune cells, possess a strong potential to inhibit inflammatory responses and promote angiogenesis in neonatal mice after cardiac injury. However, adult B cells display a more powerful pro-inflammatory and pro-fibrotic ability to exacerbate myocardial damage. Previous research showed that splenic marginal zone B lymphocytes and cardiac B lymphocytes of adult mice increased mobilization of inflammatory monocytes to the ischemic myocardium and promoted adverse post-ischemic cardiac remodeling^6,18^. Interestingly, other studies also indicated that IL-10-producing B cells enriched in adult murine pericardial adipose tissue could improve ventricular remodeling after MI by regulating monocyte migration^19,20^. Moreover, bone marrow-derived naive B lymphocytes were found to improve cardiac function after MI in adult mice^21^. These findings reveal the importance of B cell heterogeneity in ventricular remodeling outcomes post-MI. In addition to monocytes, B cells also influenced the function and number of other immune cells after cardiac injury. Adult B cell depletion inhibited the expansion of dendritic cells (DC) and T cells within pericardial adipose tissues, leading to reduced myeloid granulopoiesis and cardiac neutrophil infiltration through suppressing T cell-mediated IL-17 signaling 3 days after MI^7^. Previous research also found that adult splenic B cells were critical for liver regeneration after partial hepatectomy through secreting lymphotoxin β and mediating maintenance of IL-6-producing splenic CD169^+^ macrophages^22^. Whether neonatal cardiac B cells enhance heart regeneration by regulating the functions of other immune cells in the myocardium after myocardial injury requires further study.

In summary, our results demonstrate that cardiac B cells are required for heart regeneration in neonatal mice by promoting cardiomyocyte proliferation and injury repair. Our discovery also sheds new light on the indispensable and distinct roles of cardiac B cells and their subsets from neonatal or adult mouse heart in heart regeneration, tissue inflammation, and cardiac repair, which may provide a potential immune-based intervention strategy for cardiac recovery after heart damage.

## Methods

### Mice

Cd19-e(IRES-DTREGFP)2 mice were purchased from Shanghai Model Organisms Center Inc. The Cd19-e(IRES-DTREGFP)2 mice were generated with a transgene-driving expression of a fusion protein containing the DT receptor and the enhanced green fluorescent protein (EGFP) inserted into the *Cd19* stop codon. B cell depletion in Cd19-e(IRES-DTREGFP)2 mice was induced by intraperitoneal injection of DT (diluted in PBS, 25 ng/g body weight) three times every other day.

C57BL/6 WT mice were purchased from Shanghai JieSiJie Laboratory Animal Co., Ltd. All mice were bred and maintained in a specific pathogen-free facility with 12 h light/dark cycle and ad libitum access to standard mouse chow diet and water. All animal experiments were approved by the Scientific Investigation Committee of Tongji University in Shanghai and carried out in accordance with the Guide for the Care and Use of Laboratory Animals. All mice were euthanized by cervical dislocation after anaesthetization with an intraperitoneal injection of sodium pentobarbital (70 mg/kg body weight), and then heart tissues were dissected and detected after the operation.

### AR model of neonatal mice

The neonatal Cd19^DTR^ mice and WT control mice were subjected to anesthesia by freezing for 3–5 min, and then placed on the frozen operation table once breathing was steady. An intercostal incision was performed on the left sternal chest to expose the heart. A vacuum pump was extended into the chest cavity to suction out the heart, and then the apex was truncated with microsurgical scissors. After operation, air bubbles and blood were squeezed out of the thoracic cavity. Then mice were placed under a 37 °C heating pad to keep warm. Mice were placed back in their mothers when they woke up.

### MI model

Cd19^DTR^ mice at 2 days old were anesthetized on an ice bed. Adult Cd19^DTR^ and WT control mice (8–10 weeks old) were anesthetized by intraperitoneal injection of 1% pentobarbital sodium, and then the mouse trachea was connected to the ventilator. Subsequently, the heart was exposed and observed after lateral thoracotomy at the fourth intercostal space. A left anterior descending artery was ligated with nylon 8–0 monofilament suture (Ethicon). The rib cage and skin were closed with 5–0 and 4–0 nylon monofilament sutures respectively. Finally, mice were placed under a 37 °C heating pad to keep warm. Sham operation was performed equally involving thoracotomy, but mice did not undergo ligation of the artery.

### Masson’s trichrome staining

Masson’s trichrome staining was performed to determine collagen as reported^7^. Briefly, hearts were harvested and soaked overnight in 4% paraformaldehyde. The tissues were cut into 4 μm thick in the frozen slicer, then the slices were heated overnight at 37 °C, dewaxed, and then stained with Masson dye. Afterward, the slices were washed three times with distilled water and dehydrated with ethanol and xylene.

### Echocardiography

Cardiac function was determined by two-dimensional thoracic echocardiography using Vevo2100 (Visual Sonic VSI) and MS400 linear array transducer (38 MHz). Mice were exposed to isoflurane anesthesia during an examination. LVEF and LVFS were analyzed using Visual Sonic Vevo2100 software.

### EdU (5-ethynyl-2′-deoxyuridine) staining

Mice were injected intraperitoneally with EdU (Beyotime Biotechnology) 8 h prior to execution. Hearts were harvested and soaked overnight in 4% paraformaldehyde. The sections were cut into 4 μm thick in the frozen slicer, then the slices were permeabilized by 0.1% Triton X-100. These slices were incubated with EdU staining buffer containing click reaction buffer, CuSO4, click additive buffer, and Azide 594 for 30 min in the dark. Slices were mounted with a DAPI-containing medium and fluorescence was observed with an inverted fluorescence microscope (Leica).

### Immunostaining

Immunostaining was performed as described previously^7^. Briefly, these slices were blocked with 2% BSA, permeabilized with 0.1% Triton X-100 and then stained with the respective primary antibodies at 4 °C overnight. The following primary antibodies were used: cardiac troponin T2 (cTnT) (Abcam, ab115134, 1:100), α-actinin (Abcam, ab9465, 1:100), pH3 (Cell Signaling Technology, 9701, 1:100), Ki67 (Invitrogen, MA5-14520, 1:100). Alexa-Fluor-488- and Alexa-Fluor-594-conjugated secondary antibodies (Invitrogen, 1:200) were incubated at room temperature for 60 min in the dark.

### Publically available dataset

The following publically available dataset was used in this study.

Jin, K., et al. (GSE163465)^8^: scRNA-Seq data of two samples from the CD45^+^ immune cells in heart tissues of adult mice at day 0 and 7 after MI were downloaded from GEO and re-analyzed.

### Tissue dissociation and cell purification

Hearts were transported in a sterile culture dish with 10 ml 1 × Dulbecco’s Phosphate-Buffered Saline (DPBS, Thermo Fisher Scientific) on ice to remove the residual tissue storage solution, then minced on ice. The dissociation solution including 0.25% Trypsin (Thermo Fisher Scientific) and 10 μg/mL DNase I (Sigma) dissolved in PBS with 5% fetal bovine serum (FBS, Thermo Fisher Scientific) was used to digest heart tissues. Heart tissues were dissociated at 37 °C with a shaking speed of 50 rpm for about 40 min. The dissociated cells were repeatedly collected at intervals of 20 min to increase cell yield and viability. Cell suspensions were filtered using a 40 μm nylon cell strainer and red blood cells were removed by 1× red blood cell lysis solution (Thermo Fisher Scientific). Cell suspensions were incubated with anti-CD45 microbeads (Miltenyi Biotec) for 15 min at 4 °C, and isolated by a MACS separator. Then cells were washed with 1× DPBS containing 2% FBS. Cells were stained with 0.4% Trypan blue to check the viability on Countess II Automated Cell Counter (Thermo Fisher Scientific).

### scRNA-Seq and cell type annotations

The libraries were sequenced on NovaSeq6000 (Illumina) using 2 × 150 chemistry. scRNA-Seq data processing reads were processed using the Cell Ranger 2.1.0 pipeline with default and recommended parameters. FASTQs generated from Illumina sequencing output were aligned to the mouse genome, version GRCm38, using the STAR algorithm.

The clusters were visualized on a 2D map produced with t-distributed stochastic neighbor embedding (t-SNE). Cell types and subtypes were identified by nonlinear dimensional reduction. Cells were clustered using graph-based clustering of the PCA-reduced data with the Louvain Method. For sub-clustering, we applied the same procedure of scaled, dimensionality reduction, and clustering to the specific set of data (usually restricted to one type of cell). For each cluster, the Wilcoxon rank-sum test was used to find significant DEGs comparing the remaining clusters. Single R and known marker genes were used to identify cell types.

### Flow cytometry

To separate B cells from the heart, the heart was harvested and digested with a mixture of collagenase, DNAase and hyaluronidase. Heart tissues were dissociated at 37 °C for about 30 min. The dissociated cells were repeatedly collected at intervals of 10 min to increase cell yield and viability. Enzymatic action was stopped by adding 10% FBS and the dissociated cells were washed twice with PBS. After dissociation, the collected cell suspensions were passed through a 40 μm cell strainer. To separate B cells from the spleen, spleen tissues were ground into cell suspension by syringe. Then cell suspensions were passed through a 40 μm cell strainer. B cells in the blood were acquired from mouse orbital blood sampling. Then cell suspensions from the heart, spleen, and blood were incubated with red lysis buffer (eBioscience) for 5 min. Cells were subsequently stained with fluorochrome-conjugated antibodies against CD45 (Biolegend, 103106) and CD19 (Biolegend, 115512) in a dilution of 1:400 at room temperature for 30 min. The proportions of B cells in the heart, spleen, and blood were analyzed with CytoFlex S (Beckman Coulter).

### Quantitative PCR (Q-PCR)

Total RNA was extracted from heart tissues using the TRIzol reagent (Invitrogen). cDNAs were synthesized from total RNA by using PrimeScript RT reagent Kit with gDNA Eraser (TaKaRa) and Q-PCR was performed on the QuantStudio 6 Flex System (Thermo Fisher Scientific) with the SYBR master mix (Toyobo) following the manufacturer’s instructions. The primers for the tested mouse genes were purchased from JIELI Biology (Shanghai, China) and their sequences are as follows: *Il1β*, 5′-GGTGTGTGACGTTCCCATTAGAC-3′ (forward), 5′-CATGGAGAATATCACTTGTTGGTTGA-3′ (reverse); *Il6*, 5′-TAGTCCTTCCTACCCCAATTTCC-3′ (forward), 5′-TTGGTCCTTAGCCACTCCTTC-3′ (reverse); *Fn1*, 5′-ATGTGGACCCCTCCTGATAGT’ (forward), 5′-GCCCAGTGATTTCAGCAAAGG-3′ (reverse); *Col1α1*, 5′-GCTCCTCTTAGGGGCCACT-3′ (forward), 5′-CCACGTCTCACCATTGGGG-3′ (reverse); *Ccl5*, 5′- GCTGCTTTGCCTACCTCTCC-3′ (forward), 5′-TCGAGTGACAAACACGACTGC-3′ (reverse); *Cxcl10*, 5′-CCAAGTGCTGCCGTCATTTTC-3′ (forward), 5′-GGCTCGCAGGGATGATTTCAA-3′ (reverse); *Gapdh*, 5′-AGGTCGGTGTGAACGGATTTG-3′ (forward), 5′-TGTAGACCATGTAGTTGAGGTCA-3′ (reverse).

### Statistical analysis

All numerical data are presented as means ± SD. Statistical analysis was performed using the unpaired student’s *t*-test with data from two groups; while data from more than two groups were performed using one-way ANOVA followed by Tukey’s method for multiple comparisons. *p* < 0.05 was considered statistically significant.

### Reporting summary

Further information on research design is available in the Nature Research Reporting Summary linked to this article.

## Supplementary information

Supplementary Information

Reporting Summary

## Data Availability

Single-cell RNA transcriptome data were available in the GEO database (https://www.ncbi.nlm.nih.gov/geo/) under accession number GSE205115. All processed data supporting the findings of this study are available within the article and its supplementary information file. The non-sequencing raw data and materials are available from the corresponding author on reasonable request.
