# Supplementary material for: Murine neonatal cardiac B cells promote cardiomyocyte proliferation and heart regeneration
Source: NPJ Regen Med. 2023 Feb 11;8:7. doi: 10.1038/s41536-023-00282-7 (PMC9922252; doi:10.1038/s41536-023-00282-7)
Supplement: Supplementary file 1 — Supplementary Information [file 41536_2023_282_MOESM1_ESM.pdf]

# Supplementary Information

## Murine neonatal cardiac B cells promote cardiomyocyte proliferation and heart regeneration

Yong Tan, Xuewen Duan, Bo Wang, Xingguang Liu, Zhenzhen Zhan

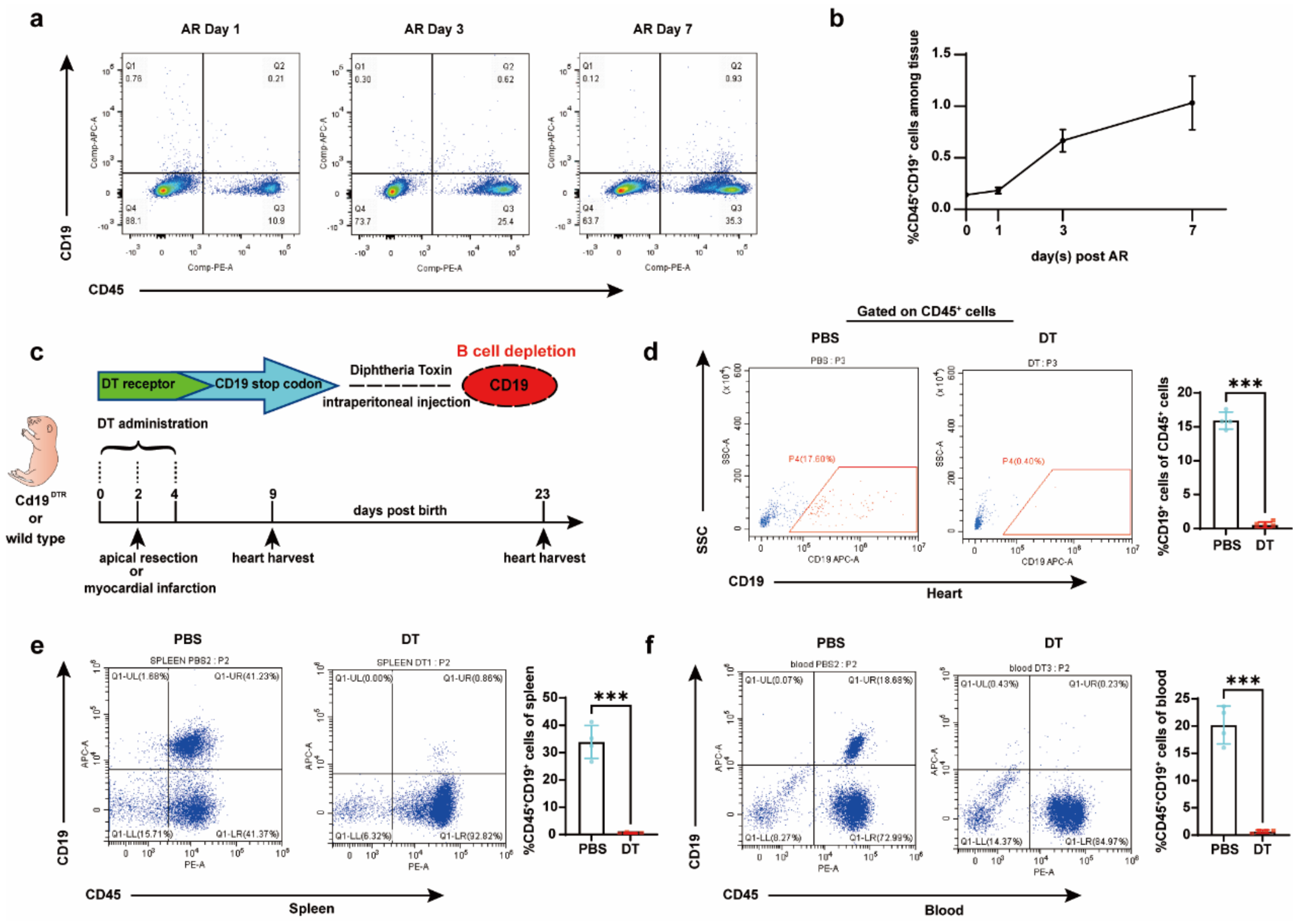


**Supplementary Figure 1. The proportion of cardiac B cells gradually increases at day 1–7 after AR.** **a** Representative FACS analysis of $CD45^+CD19^+$ B cells in heart tissues of P1 WT mice at the indicated days after AR. **b** Quantification of the relative proportion of $CD45^+CD19^+$ cells in the harvested heart tissues at day 0, 1, 3 and 7 after AR. The proportion of cardiac B cells at day 0 represented the proportion of B cells in mouse heart with sham operation (n = 3 mice for each group). **c** Schematic diagram showing the experimental design. **d-f** Representative FACS analysis of $CD19^+$ B cells among cardiac $CD45^+$ cells (**d**) and $CD45^+CD19^+$ B cells from spleen (**e**) or blood (**f**)

of adult $CD19^{DTR}$ mice with DT or PBS administration. Each symbol in quantification represents one mouse (n = 4 mice for each group; mean ± SD; unpaired Student's t-test). ***$p < 0.001$.

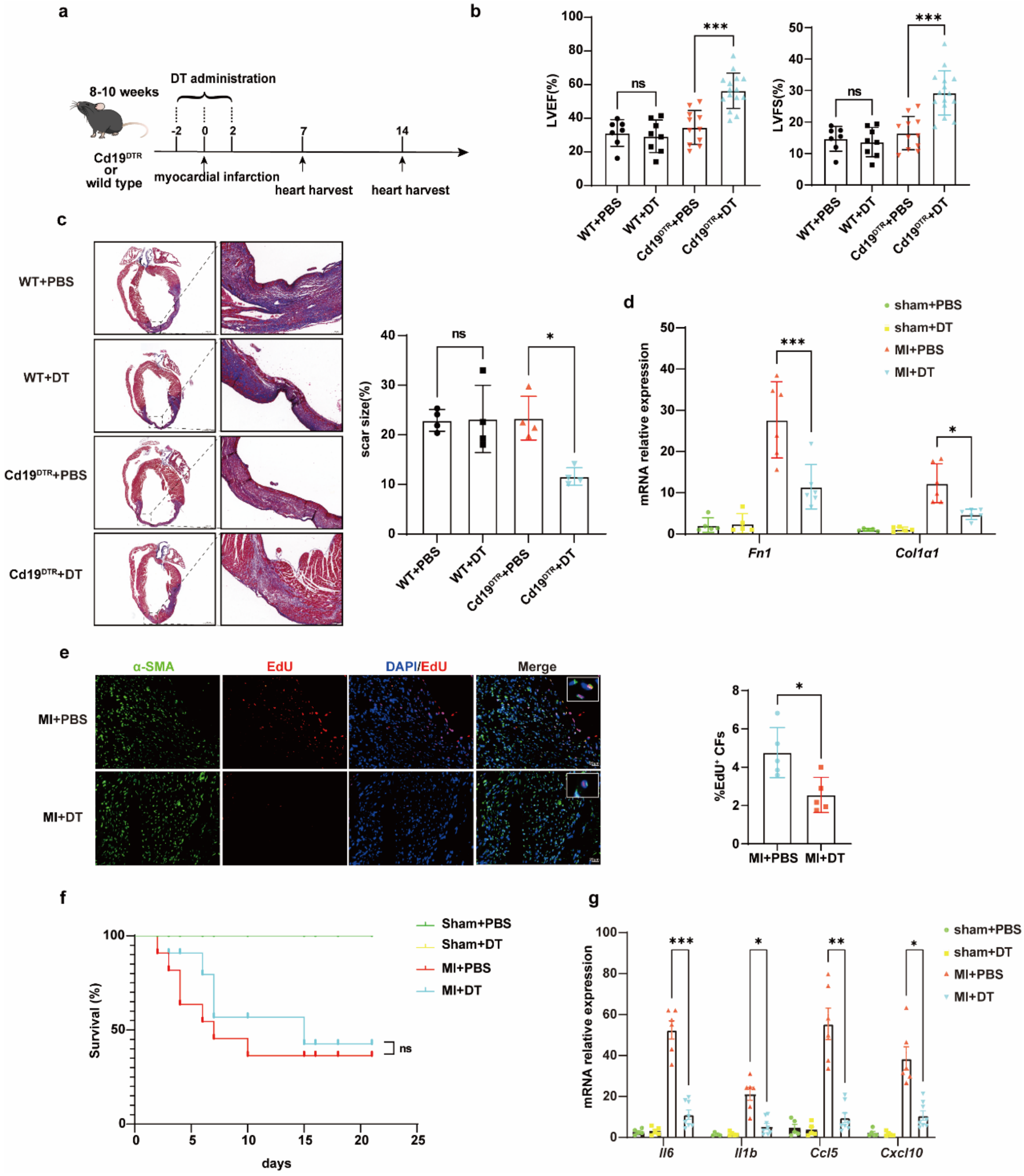


**Supplementary Figure 2. Cardiac B cells of adult mice exacerbate tissue injury after MI by promoting inflammation and fibrosis**. **a** Schematic diagram showing the experimental design. **b** Echocardiographic measurement of LVEF and LVFS of adult

CD19$^{DTR}$ or WT control mice with DT or PBS administration at day 7 after MI. Each symbol in quantification represents one mouse (WT + PBS, n = 7 mice; WT + DT, n = 8 mice; CD19$^{DTR}$ + PBS, n = 11 mice; CD19$^{DTR}$ + DT, n = 15 mice, one-way ANOVA). **c** Representative images and quantification of heart sections stained with Masson's trichrome from adult CD19$^{DTR}$ or WT control mice with DT or PBS administration at day 7 after MI. Each symbol in quantification represents one mouse (n = 4 mice for each group, one-way ANOVA). Scale bars, 1000 μm (left) or 100 μm (right). **d** Q-PCR analysis of *Fn1* and *Col1α1* mRNA expression in myocardial tissues from adult CD19$^{DTR}$ mice with DT or PBS administration at day 14 after MI or sham operation. Each symbol in quantification represents one mouse (sham + PBS, n = 5 mice; sham + DT, n = 5 mice; MI + PBS, n = 6 mice; MI + DT, n = 6 mice, one-way ANOVA). **e** Representative images and quantification of EdU$^{+}$ cardiac fibroblasts from myocardial tissues of adult CD19$^{DTR}$ mice with DT or PBS administration at day 7 post-MI. Each symbol in quantification indicates one representative image from one heart section of one mouse (n = 5 mice for each group, unpaired Student's t-test). Scale bars, 20 μm. **f** Kaplan-Meier survival curves show the survival of CD19$^{DTR}$ mice with DT or PBS administration subjected to sham operation or MI (n = 12 mice for each group, log-rank test). **g** Q-PCR analysis of *Il6*, *Il1b*, *Ccl5* and *Cxcl10* mRNA expression in myocardial tissues from adult CD19$^{DTR}$ mice with DT or PBS administration at day 4 after MI or sham operation. Each symbol in quantification represents one mouse (sham + PBS, n = 5 mice; sham + DT, n = 5 mice; MI + PBS, n = 6 mice; MI + DT, n = 8 mice; one-way ANOVA). The data were shown as mean ± SD (**b**-**e**, **g**). $*p < 0.05$, $**p < 0.01$, $***p <$

0.001.

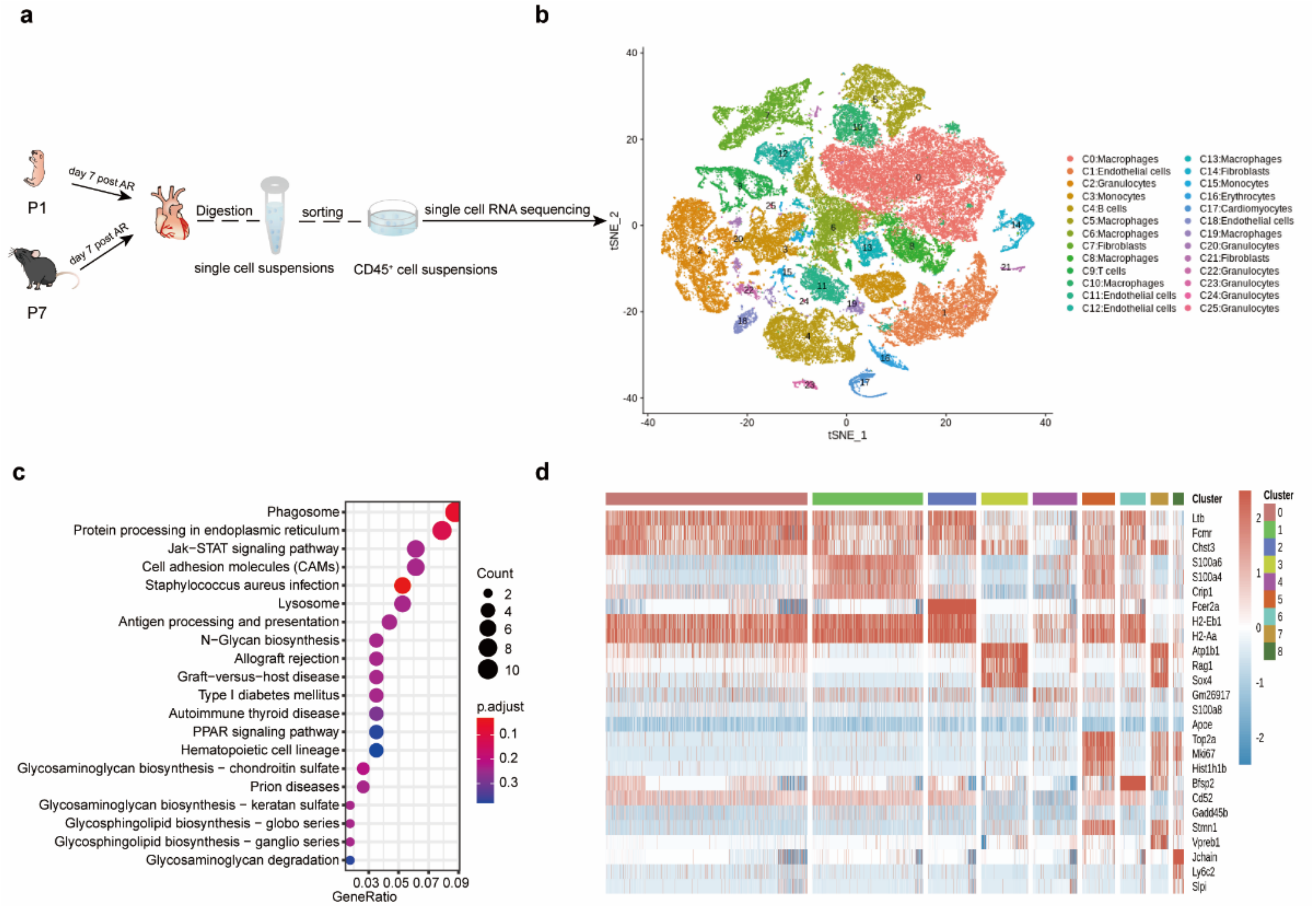


**Supplementary Figure 3. Single-cell atlas reveals heterogeneity of cardiac B cells.** **a** Schematic diagram showing the experimental design for scRNA-Seq of CD45$^+$ cells from heart tissues at day 7 after AR operated on P1 and P7 WT mice. **b** Twenty-six distinct cell clusters identified in heart tissues from P1 and P7 WT mice were visualized by t-SNE plotting, with each cell color-coded for its associated cluster. **c** KEGG enrichment analysis of differentially expressed genes in B cells of P1 WT mouse hearts compared with that in P7 hearts at day 7 after AR. **d** Heatmap diagram showing the key marker genes in nine phenotypes of B cells in heart tissues at day 7 after AR operated

on P1 and P7 WT mice or from adult mouse heart after MI (GSE163465).
