# Supplementary material for: Murine neonatal cardiac B cells promote cardiomyocyte proliferation and heart regeneration
Source: NPJ Regen Med. 2023 Feb 11;8:7. doi: 10.1038/s41536-023-00282-7 (PMC9922252; doi:10.1038/s41536-023-00282-7)
Supplement: Supplementary file 2 — Reporting Summary [file 41536_2023_282_MOESM2_ESM.pdf]

nature portfolio

Corresponding author(s): Zhenzhen Zhan; NPJREGENMED-01073R3

Last updated by author(s): Jan 14, 2023

Reporting Summary

Nature Portfolio wishes to improve the reproducibility of the work that we publish. This form provides structure for consistency and transparency in reporting. For further information on Nature Portfolio policies, see our Editorial Policies and the Editorial Policy Checklist.

Statistics

For all statistical analyses, confirm that the following items are present in the figure legend, table legend, main text, or Methods section.

n/a	Confirmed	
☐	☒	The exact sample size (*n*) for each experimental group/condition, given as a discrete number and unit of measurement
☐	☒	A statement on whether measurements were taken from distinct samples or whether the same sample was measured repeatedly
☐	☒	The statistical test(s) used AND whether they are one- or two-sided<br>*Only common tests should be described solely by name; describe more complex techniques in the Methods section.*
☐	☒	A description of all covariates tested
☐	☒	A description of any assumptions or corrections, such as tests of normality and adjustment for multiple comparisons
☐	☒	A full description of the statistical parameters including central tendency (e.g. means) or other basic estimates (e.g. regression coefficient) AND variation (e.g. standard deviation) or associated estimates of uncertainty (e.g. confidence intervals)
☐	☒	For null hypothesis testing, the test statistic (e.g. *F*, *t*, *r*) with confidence intervals, effect sizes, degrees of freedom and *P* value noted<br>*Give P values as exact values whenever suitable.*
☒	☐	For Bayesian analysis, information on the choice of priors and Markov chain Monte Carlo settings
☒	☐	For hierarchical and complex designs, identification of the appropriate level for tests and full reporting of outcomes
☒	☐	Estimates of effect sizes (e.g. Cohen's *d*, Pearson's *r*), indicating how they were calculated

Our web collection on statistics for biologists contains articles on many of the points above.

Software and code

Policy information about availability of computer code

Data collection	microscope: Leica DMI3000 B fluorescence microscope, Leica application suite v3.8.<br>Ecocardiography: Vevo2100 (Visual Sonic VSI, Toronto, Canada).<br>q-PCR: QuantStudio Real-Time PCR System (Applied Biosystems)<br>scRNA-seq: NovaSeq6000 (Illumina)<br>Flow cytometry: CytoFlex S (Beckman Coulter)
Data analysis	scRNA-seq analysis: R(v3.5.3), DEGs("FindAllMarkers"), enrichment analysis (v3.14.3), Seurat (v3.2.2)<br>Quantification of IF: Image J(v 1.51m9)<br>Statistical analysis for echocardiographic data, histological quantification and qPCR: GraphPad Prism 8.<br>Flow cytometry analysis: CytExpert

For manuscripts utilizing custom algorithms or software that are central to the research but not yet described in published literature, software must be made available to editors and reviewers. We strongly encourage code deposition in a community repository (e.g. GitHub). See the Nature Portfolio guidelines for submitting code & software for further information.

Data

Policy information about availability of data

All manuscripts must include a data availability statement. This statement should provide the following information, where applicable:

- Accession codes, unique identifiers, or web links for publicly available datasets
- A description of any restrictions on data availability
- For clinical datasets or third party data, please ensure that the statement adheres to our policy

Single-cell RNA transcriptome data are available in the GEO database (https://www.ncbi.nlm.nih.gov/geo/) under accession number GSE205115.
The raw data that support the findings of this study are available from the corresponding author, Zhenzhen Zhan, upon reasonable request.

Human research participants

Policy information about studies involving human research participants and Sex and Gender in Research.

Reporting on sex and gender	*Use the terms sex (biological attribute) and gender (shaped by social and cultural circumstances) carefully in order to avoid confusing both terms. Indicate if findings apply to only one sex or gender; describe whether sex and gender were considered in study design whether sex and/or gender was determined based on self-reporting or assigned and methods used. Provide in the source data disaggregated sex and gender data where this information has been collected, and consent has been obtained for sharing of individual-level data; provide overall numbers in this Reporting Summary. Please state if this information has not been collected. Report sex- and gender-based analyses where performed, justify reasons for lack of sex- and gender-based analysis.*
Population characteristics	*Describe the covariate-relevant population characteristics of the human research participants (e.g. age, genotypic information, past and current diagnosis and treatment categories). If you filled out the behavioural & social sciences study design questions and have nothing to add here, write "See above."*
Recruitment	*Describe how participants were recruited. Outline any potential self-selection bias or other biases that may be present and how these are likely to impact results.*
Ethics oversight	*Identify the organization(s) that approved the study protocol.*

Note that full information on the approval of the study protocol must also be provided in the manuscript.

Field-specific reporting

Please select the one below that is the best fit for your research. If you are not sure, read the appropriate sections before making your selection.

☒ Life sciences ☐ Behavioural & social sciences ☐ Ecological, evolutionary & environmental sciences

For a reference copy of the document with all sections, see nature.com/documents/nr-reporting-summary-flat.pdf

Life sciences study design

All studies must disclose on these points even when the disclosure is negative.

Sample size	Sample size in our experiments was determined based on standards in the field and experiments to obtain statistical significance and reproducibility. At least triplicates were used to meet the minimal requirements for statistical analysis and the detailed sample size was demonstrated in the figure legends.
Data exclusions	No data were excluded from the analyses.
Replication	Replicates were used in in vivo experiments as noted in figure legends. qPCR and flow cytometry experiments were performed at least three times independently. All attempts at replication were successful.
Randomization	All animals were randomly assigned to the experimental groups.
Blinding	Whenever possible, data were analyzed blind to condition. Data that require an automated analysis pipeline were not analyzed blind to conditions, including scRNA-seq data.

Reporting for specific materials, systems and methods

We require information from authors about some types of materials, experimental systems and methods used in many studies. Here, indicate whether each material, system or method listed is relevant to your study. If you are not sure if a list item applies to your research, read the appropriate section before selecting a response.

Materials & experimental systems

n/a	Involved in the study
☐	☒ Antibodies
☒	☐ Eukaryotic cell lines
☒	☐ Palaeontology and archaeology
☐	☒ Animals and other organisms
☒	☐ Clinical data
☒	☐ Dual use research of concern

Methods

n/a	Involved in the study
☒	☐ ChIP-seq
☐	☒ Flow cytometry
☒	☐ MRI-based neuroimaging

Antibodies

Antibodies used

Primary antibodies used in Immunofluorescence experiments:
1. Monoclonal Anti-α-Actinin (Sarcomeric) antibody produced in mouse (Sigma, A7811)
2. Anti-Cardiac Troponin T antibody (abcam, ab115134)
3. Ki-67 Recombinant Rabbit Monoclonal Antibody (SP6) (invitrogen, MA5-14520)
4. Phospho-Histone H3 (Ser10) Antibody (cell signaling technology, 9701S)
5. Anti-alpha smooth muscle Actin antibody (abcam, ab5694)
Primary antibodies used in flow cytometry experiments:
1. PE anti-mouse CD45 Antibody (Biolegend, 103106)
2. APC anti-mouse CD19 Antibody (Biolegend, 115512)

Validation

All following primary antibodies used in this study were validated and were cited in the litterature.
1. Monoclonal Anti-α-Actinin (Sarcomeric) antibody produced in mouse. https://www.sigmaaldrich.cn/CN/en/product/sigma/a7811
Ref: Adenylyl cyclase subtype-specific compartmentalization: differential regulation of L-type Ca2+ current in ventricular myocytes. Valeriy Timofeyev et al. Circulation research, 112(12), 1567-1576 (2013-04-24)
2. Anti-Cardiac Troponin T antibody. https://www.abcam.com/cardiac-troponin-t-antibody-ab115134.html
Ref: Behavioral variation according to feeding organ diversification in glossiphoniid leeches (Phylum: Annelida). Kwak HJ et al. Sci Rep 11:10940 (2021).
3. Ki-67 Recombinant Rabbit Monoclonal Antibody (SP6). https://www.thermofisher.cn/cn/en/antibody/product/Ki-67-Antibody-clone-SP6-Recombinant-Monoclonal/MA5-14520
Ref: High Fat Diets Induce Colonic Epithelial Cell Stress and Inflammation that is Reversed by IL-22. Gulhane, M., et al. Scientific reports, 6, 28990 (2016).
4. Phospho-Histone H3 (Ser10) Antibody. https://www.cellsignal.com/products/primary-antibodies/phospho-histone-h3-ser10-antibody/9701
Ref: The Mir181ab1 cluster promotes KRAS-driven oncogenesis and progression in lung and pancreas. Karmele Valencia, et. al. J Clin Invest; 130(4):1879-1895 (2020).
5. Anti-alpha smooth muscle Actin antibody. https://www.abcam.cn/alpha-smooth-muscle-actin-antibody-ab5694.html
Ref: Interleukin-17A derived from mast cells contributes to fibrosis in gastric cancer with peritoneal dissemination. Gunjigake K et al. Gastric Cancer 24:31-44 (2021).
6. PE anti-mouse CD45 Antibody. https://www.biolegend.com/en-us/products/pe-anti-mouse-cd45-antibody-100
Ref: Single-cell analysis reveals T cell infiltration in old neurogenic niches. Ben W Dulken, et al. Nature 571(7764):205-210 (2019).
7. APC anti-mouse CD19 Antibody. https://www.biolegend.com/en-us/products/apc-anti-mouse-cd19-antibody-1526
Ref: T Follicular Helper Cell-Dependent Clearance of a Persistent Virus Infection Requires T Cell Expression of the Histone Demethylase UTX. Kevin D Cook, et al. Immunity 20;43(4):703-14 (2015).

Animals and other research organisms

Policy information about studies involving animals; ARRIVE guidelines recommended for reporting animal research, and Sex and Gender in Research

Laboratory animals

Male C57BL/6J mice (8-10 weeks), Female and Male C57BL/6J mice (P2 old). The Cd19-e(IRES-DTREGFP)2 mice were generated with a transgene driving expression of a fusion protein containing the DT receptor and the enhanced green fluorescent protein (EGFP) inserted into Cd19 stop codon. B cells depletion in Cd19-e(IRES-DTREGFP)2 mice was induced by intraperitoneal injection of DT (diluted in PBS, 25ng/g body weight) 3 times every other day. Male Cd19-e(IRES-DTREGFP)2 mice (8-10 weeks), Female and Male Cd19-e(IRES-DTREGFP)2 mice (P2 old).

Wild animals

not involved

Reporting on sex

not involved

Field-collected samples

not involxed

Ethics oversight

All of the animal experiments were approved by the Animal Care and Use Committee of Tongji University School of Medicine.

Note that full information on the approval of the study protocol must also be provided in the manuscript.

Flow Cytometry

Plots

Confirm that:

☒ The axis labels state the marker and fluorochrome used (e.g. CD4-FITC).

☒ The axis scales are clearly visible. Include numbers along axes only for bottom left plot of group (a 'group' is an analysis of identical markers).

☐ All plots are contour plots with outliers or pseudocolor plots.

☒ A numerical value for number of cells or percentage (with statistics) is provided.

Methodology

Sample preparation	To separate B cells from the heart, the heart was harvested and digested with a mixture of collagenase, DNAase and hyaluronidase. Heart tissues were dissociated at 37 ℃ for about 30 min. The dissociated cells were repeatedly collected at interval of 10 min to increase cell yield and viability. Enzymatic action was stopped by adding 10% FBS and the dissociated cells were washed twice with PBS. After dissociation, the collected cell suspensions were passed through a 40 µm cell strainer. To separate B cells from the spleen, spleen tissues were ground into cell suspension by syringe. Then cell suspensions were passed through a 40 µm cell strainer. B cells in the blood were acquired from mouse orbital blood sampling. Then cell suspensions from heart, spleen and blood were incubated with red lysis buffer (eBioscience) for 5 min. Cells were subsequently stained with fluorochrome-conjugated antibodies against CD45 and CD19 (Biolegend) in a dilution of 1:400 at room temperature for 30 min. The proportions of B cells in heart, spleen and blood were analyzed with CytExpert.
Instrument	CytoFlex S (Beckman Coulter)
Software	Flow cytometry analysis: CytExpert
Cell population abundance	No sorting was used in this manuscript.
Gating strategy	Doublet discrimination was performed by FSC-A vs FSC-H followed by removal of debris. Immune cells (CD45 positive cells) were gated on SSC/FSC plots. B cells were gated according to the result of CD19 staining. Gating was determined by blank and single color-staining.

☒ Tick this box to confirm that a figure exemplifying the gating strategy is provided in the Supplementary Information.
